# Differences between the most used equations in BAT-human studies to estimate parameters of skin temperature in young lean men

**DOI:** 10.1038/s41598-017-10444-5

**Published:** 2017-09-05

**Authors:** Borja Martinez-Tellez, Guillermo Sanchez-Delgado, Francisco M. Acosta, Juan M. A. Alcantara, Mariëtte R. Boon, Patrick C. N. Rensen, Jonatan R. Ruiz

**Affiliations:** 1 PROFITH “PROmoting FITness and Health through physical activity” research group. Department of Physical Education and Sport, Faculty of Sport Sciences, University of Granada, Granada, Spain; 2 Department of Medicine, Division of Endocrinology, and Einthoven Laboratory for Experimental Vascular Medicine, Leiden University Medical Center, Leiden, The Netherlands

## Abstract

Cold exposure is necessary to activate human brown adipose tissue (BAT), resulting in heat production. Skin temperature is an indirect measure to monitor the body’s reaction to cold. The aim of this research was to study whether the most used equations to estimate parameters of skin temperature in BAT-human studies measure the same values of temperature in young lean men (n = 11: 23.4 ± 0.5 years, fat mass: 19.9 ± 1.2%). Skin temperature was measured with 26 ibuttons at 1-minute intervals in warm and cold room conditions. We used 12 equations to estimate parameters of mean, proximal, and distal skin temperature as well as skin temperature gradients. Data were analysed with Temperatus software. Significant differences were found across equations to measure the same parameters of skin temperature in warm and cold room conditions, hampering comparison across studies. Based on these findings, we suggest to use a set of 14 ibuttons at anatomical positions reported by ISO STANDARD 9886:2004 plus five ibuttons placed on the right supraclavicular fossa, right middle clavicular bone, right middle upper forearm, right top of forefinger, and right upper chest.

## Introduction

In 1937, Hardy and Du Bois^1^ studied the effect of different environment exposures (cold and heat) over parameters of skin temperature. Thereafter, parameters of skin temperature and thermal responses were studied as thermal-physiological responses to different stimulus such as exercise^2^, anaesthesia-treatment^3^, circadian-rythms^4^, or heat^5^ and cold^6^ environment.

Since 2009, cold exposure is used in human studies as one of the main activators of brown adipose tissue (BAT) before performing a ^18^F-Fluorodeoxiglucose Positron emission tomography/computed tomography (^18^F-FDG-PET/CT)^7–12^. BAT is highly regulated by the sympathetic nervous system (SNS) increasing body temperature when mammals are exposed to cold^13^. Therefore, skin temperature measurements could play an important role as a possible indirect marker of BAT activity or volume^14–17^.

To date, consensus about which equations are better to estimate parameters of mean, proximal, or distal skin temperature and body-gradients is non-existent. Moreover, whereas some human BAT studies^7, 14, 18–23^ used different equations to estimate the same parameters of skin temperature, other studies did not report the equations used^15, 24, 25^. This fact hampers comparability between studies. The lack of knowledge about a set of validated equations or alternative instruments as “gold-standard” to measure skin temperature hinders comparisons between studies. Of note is that most of the human-studies used ibuttons (a valid^26, 27^ and reliable^27^ tool) to measure skin temperature.

Most of the equations used in the cooling protocols before BAT activation were not validated (i.e. mean, proximal, distal skin temperature, and body gradients) against any “gold-standard”, neither were most of the body-gradients used. To note is, however, that supraclavicular skin temperature^14, 15, 28^ and a supraclavicular gradient^29^ were validated against ^18^F-FDG-PET/CT and were postulated as an indirect measurement of BAT volume and activity. Sessler *et al*.^3^ validated a peripheral skin temperature gradient (see Table 1) as a proxy of peripheral vasoconstriction measured by laser Doppler monitor. Furthermore, there are some thermophysiological models used to predict local skin temperature^25, 30, 31^, yet these models need to be applied in the specific cooling protocols used in the BAT activation.

**Table 1:** Equations used to estimate parameters of skin temperature.

Outcome	Reference	ibuttons (n)	Anatomical positions. Fig. 1	Participants (n)	Equations
Mean skin temperature	14 ISO 9886–2004^41^ *(14-ISO)*	14	From 1 to 14	9	(Forehead*0.07) + (Neck*0.07) + (Right Scapula*0.07) + (Left Chest*0.07) + (Right Deltoid*0.07) + (Left Elbow*0.07) + (Right Abdomen*0.07) + (Left Hand*0.07) + (Left Lumbar *0.07) + (Right Thigh*0.07) + (Left Hamstring*0.07) + (Right Shinbone*0.07) + (Left Gastrocnemius*0.07) + (Right Instep*0.07)
	8 ISO 9886–2004^41^ *(8-ISO)*	8	1, 3, 4, 5, 6, 9, 10, 13	10	(Forehead*0.07) + (Right Scapula*0.175) + (Left Chest*0.175) + (Right Deltoid*0.07) + (Left Elbow*0.07) + (Left Hand*0.05) + (Right Thigh*0.19) + (Left Gastrocnemius*0.2)
	4 ISO 9886–2004^41^ *(4-ISO)*	4	2, 3, 9, 12	10	(Neck*0.28) + (Right Scapula*0.28) + (Left Hand*0.16) + (Right Shinbone*0.28)
	Boon *et al*.^15^ *(Boon)*	5	10, 16, 8, 9, 14	10	(((Right Thigh*0.383) + (Right Clavicular *0.293) + (Right Abdomen*0.324)) + ((Left Hand + Right Instep)/2))/4)
	*PROFITH*	26	From 1 to 26	8	(Forehead + Neck + Right Scapula + Left Chest + Right Deltoid + Left Elbow + Right Abdomen + Left Hand + Left Lumbar + Right Thigh + Left Hamstring + Right Shinbone + Left Gastrocnemius + Right Instep + Right Supraclavicular + Right Clavicular + Right Subclavicular + Left Forearm + Left top of forefinger + Right Forearm + Right top of forefinger + Left Shinbone + Right Gastrocnemius + Left Instep + Right Chest + Left Thigh)/26
Proximal skin temperature	Kräuchi *et al*.^36^ *(Kräuchi)*	4	1, 10, 17, 8	10	(Forehead*0.093) + (Right Thigh*0.347) + (Right Subclavicular*0.266) + (Right Abdomen*0.294)/4
	van Marken Lichtenbelt *et al*.^26^ *(Van Marken)*	3	10, 17, 8	10	(Right Thigh*0.383) + (Right Subclavicular*0.293) + (Right Abdomen*0.324)
	Schellen *et al*.^32^ *(Schellen)*	4	3, 7, 4, 8	11	(Right Scapula + Left Lumbar + Left Chest + Right Abdomen)/4
	Boon *et al*.^15^ *(Boon)*	3	10, 16, 8	10	(Right Thigh*0.383) + (Right Clavicular*0.293) + (Right Abdomen*0.324)
	*PROFITH*	5	8, 25, 4, 3, 2	11	(Right Abdomen + Right Chest + Left Chest + Right Scapula + Neck)/5
Distal skin temperature	Kräuchi *et al*.^36^ *(Krauchi)*	2	9, 14	11	(Left Hand + Right Instep)/2
	*PROFITH*	6	1, 19, 21, 14, 24, 9	11	(Forehead + Left top of forefinger + Right top of forefinger + Right Instep + Left Instep + Left Hand)/6
Body temperature gradient	Boon *et al*.^15^ *(Boon)*	5	9, 14, 10, 16, 8	10	[(Left Hand + Right Instep)/2]- [(Right Thigh*0.383) + (Right Clavicular*0.293) + (Right Abdomen*0.324)]
	*PROFITH*	11	1, 19, 21, 14, 24, 9, 8, 25, 3, 2	11	[(Forehead + Left top of forefinger + Right top of forefinger + Right Instep + Left Instep + Left Hand)/6]-[(Right Abdomen + Right Chest + Left Chest + Right Scapula + Neck)/5]
Supraclavicular temperature gradient	*PROFITH: (S-LC)*	2	15, 4	11	(Right Supraclavicular(S)-Left Chest(LC))
	Lee *et al*.^29^ *(Lee S-RC)*	2	15, 25	11	(Right Supraclavicular(S)- Right Chest (RC))
	*PROFITH: (S-SB)*	2	15, 17	11	(Right Supraclavicular(S)-Right Subclavicular(SB))
Peripheral temperature Gradient	Sessler *et al*.^3^ Right arm	2	20, 21	11	(Right Forearm-Right Top of forefinger)
	PROFITH: Left arm	2	18, 19	11	(Left Forearm-Left Top of forefinger)
	PROFITH: Right Leg	2	23, 14	11	(Right Gastrocnemius- Right Instep)
	PROFITH: Left Leg	2	13, 24	11	(Left Gastrocnemius-Left Instep)

Taking into account the lack of consensus on which equation to use and the high discrepancy that exists across studies that measured skin temperature in response to cold exposure, we studied if the most used equations to estimate parameters of skin temperature in human BAT studies measure the same in young lean men.

## Methods

A total of 11 men (23.4 ± 0.5 years) took part in the present study. All participants were healthy, lean (fat mass: 19.9 ± 1.2%) (see Table 2), non-smokers, and were not taking any medication that could have altered the cardiovascular or thermoregulatory responses to cold exposure. The study protocol and informed consent followed the Declaration of Helsinki (revision of 2013). This study was approved by The Human Research Ethics Committee of the University of Granada (n°924) and by the Servicio Andaluz de Salud (Centro de Granada, CEI-Granada).

**Table 2:** Characteristics of the participants.

	Mean		SE
Age (years)	23.4	±	0.5
Body mass index (kg/m^2^)	23.2	±	0.4
Fat free mass (kg)	58.2	±	8.8
Fat mass (kg)	13.8	±	0.9
Fat mass (%)	19.9	±	1.2
Bone mineral density (g/cm^2^)	1.31	±	0.03

SE: Standard error.

### Previous conditions to the study day

The study was conducted between March and April 2016 in Granada (Southern Spain). Participants arrived at the research centre by bus or by car in fasted condition (at least 8 hours after their last meal), between 8 a.m. and 4 p.m. They were advised to refrain from any type of physical activity or exercise in the 48 hours prior to the study day. Additionally, participants were required not to drink alcoholic or caffeine-containing beverages in the 24 hours prior to the study day.

### Skin temperature registration

**Figure 1:**
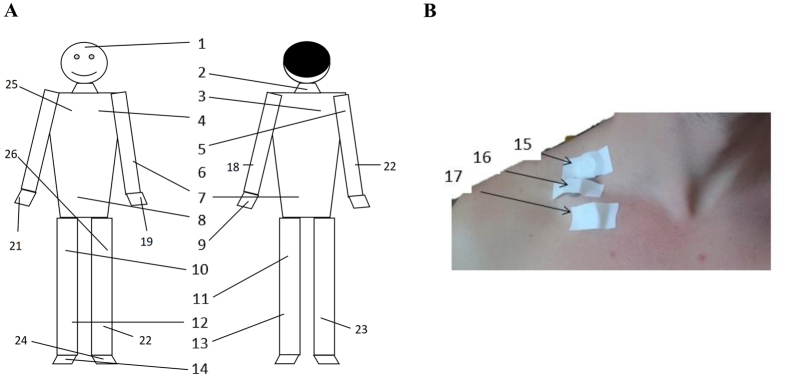
Anatomical position of 26 ibuttons. (**A**) Distribution of the ibuttons over the body. (**B**) Distribution of the ibuttons on the right clavicular sites.

We measured skin temperature with 26 ibuttons^32^ (DS-1922 L, Thermochron; resolution: 0.0625 °C; Maxim, Dallas, USA). Ibuttons are valid and reliable devices to measure skin temperature in humans^26, 27^. We attached the ibuttons to the skin with adhesive tape (Fixomull, Beiersdorf AG, Hamburg, Germany) on different body sites (see Fig. 1)^14, 26, 32–36^. Skin temperature was recorded at 1-minute intervals. We reviewed which skin temperature equations were most used in human BAT studies^7, 14, 18–23^, and selected 12 equations to estimate parameters of skin temperature including mean, proximal, and distal skin temperature (Table 1). Moreover, we calculated the gradient over forearms minus top of forefingers as a measure of peripheral vasoconstriction in the arms^37^, as well as the heat loss capacity of the supraclavicular zone^29^ and of the whole body through different body gradients^15^. Taking into account the number of ibuttons used in the present study (n = 26), we computed 6 new equations using the highest number of ibuttons possible to estimate parameters of skin temperature (see PROFITH equations, Table 1). All data recorded by the devices and equations were analysed by the Temperatus software (http://profith.ugr.es/temperatus).

### Cooling protocol

**Figure 2:**
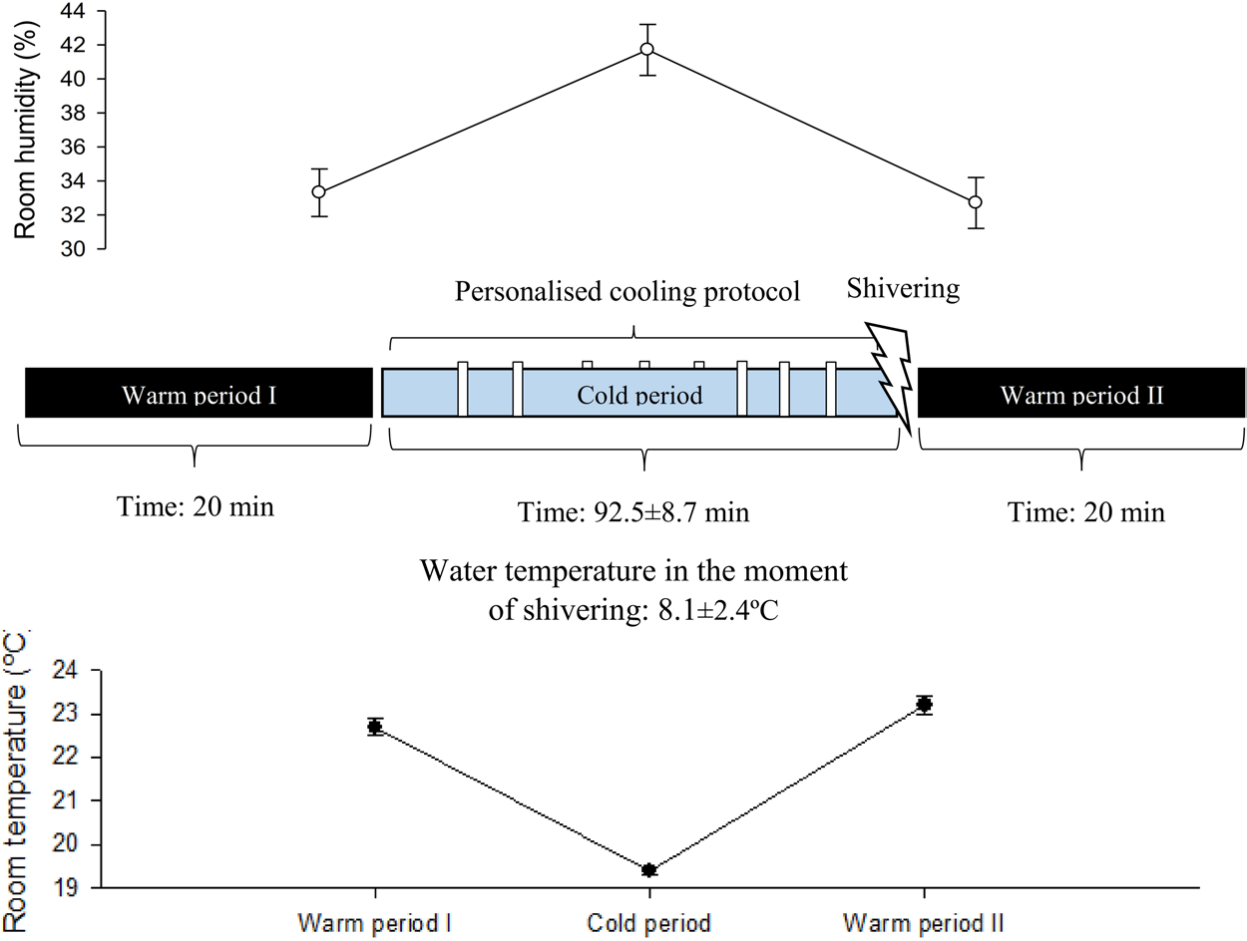
Cooling protocol. Each vertical white bars represents a decrease of the water temperature of the cooling vest. Upper graphic represents mean room humidity during warm period I, cold period, warm period II. Lower graphics represents mean room temperature during warm period I, cold period, warm period II.

Participants were barefoot and wore a short standardized T-shirt [clo-value: 0.20^38, 39^]. We controlled the room temperature (see Fig. 2), and we avoided potential airflow in the room. The participants lay on a bed for 20 minutes in a warm room (warm period I, 22.7 ± 0.2 °C). Thereafter, participants were moved into a cold room (19.4 ± 0.1 °C) where we applied a cooling protocol until shivering occurred (Fig. 2). In the cold room, the participants lay on a bed for 15 minutes, after which they were equipped with a temperature-controlled water circulation-cooling vest (Polar Products Inc., Ohio, USA)^40^. The cooling vest covered the individuals’ clavicular region, as well as the chest, abdomen, and back. The water temperature started at 17 °C and decreased progressively (~1 °C) every 10 minutes until shivering occurred (cold period mean time: 92.5 ± 8.7 minutes; water mean temperature of shivering: 8.1 ± 2.41 °C, see Fig. 2). We determined shivering both visually and by asking the participants if they were experiencing shivering. Shivering was confirmed by EMG in 6 participants. Once shivering was determined, the participants returned to the warm room (23.2 ± 0.2 °C) and lay on the bed for another 20 minutes (warm period II) without the cooling vest. The participants were not allowed to move on the bed, read or watch a film, or to be covered by a blanket or a sheet. At the end of the study day, we measured body composition by Dual Energy X-ray Absorptiometry scan (HOLOGIC, QDR 4500 W).

### Statistical Analysis

Data are presented as mean and standard error. The skin temperature data were taken as average in the last five minutes of the warm period I (before initializing cooling protocol), in the last five minutes of the warm period II, and in the five minutes prior to shivering (cold period). We excluded the data from the equations in the analysis when at least one ibutton failed during the measurements (see Table 1). We analysed differences across the study equations using analysis of variance (ANOVA) with Bonferroni adjustments for post-hoc comparisons, by periods (warm period I, cold period, and warm period II). We compared mean differences of skin temperature across temperature conditions, using ANOVA for repeated measurements. All the analyses were conducted using the Statistical Package for Social Sciences (SPSS, v. 22.0, IBM SPSS Statistics, IBM Corporation), and the level of significance was set to < 0.05.

## Results

### Warm period I

**Figure 3:**
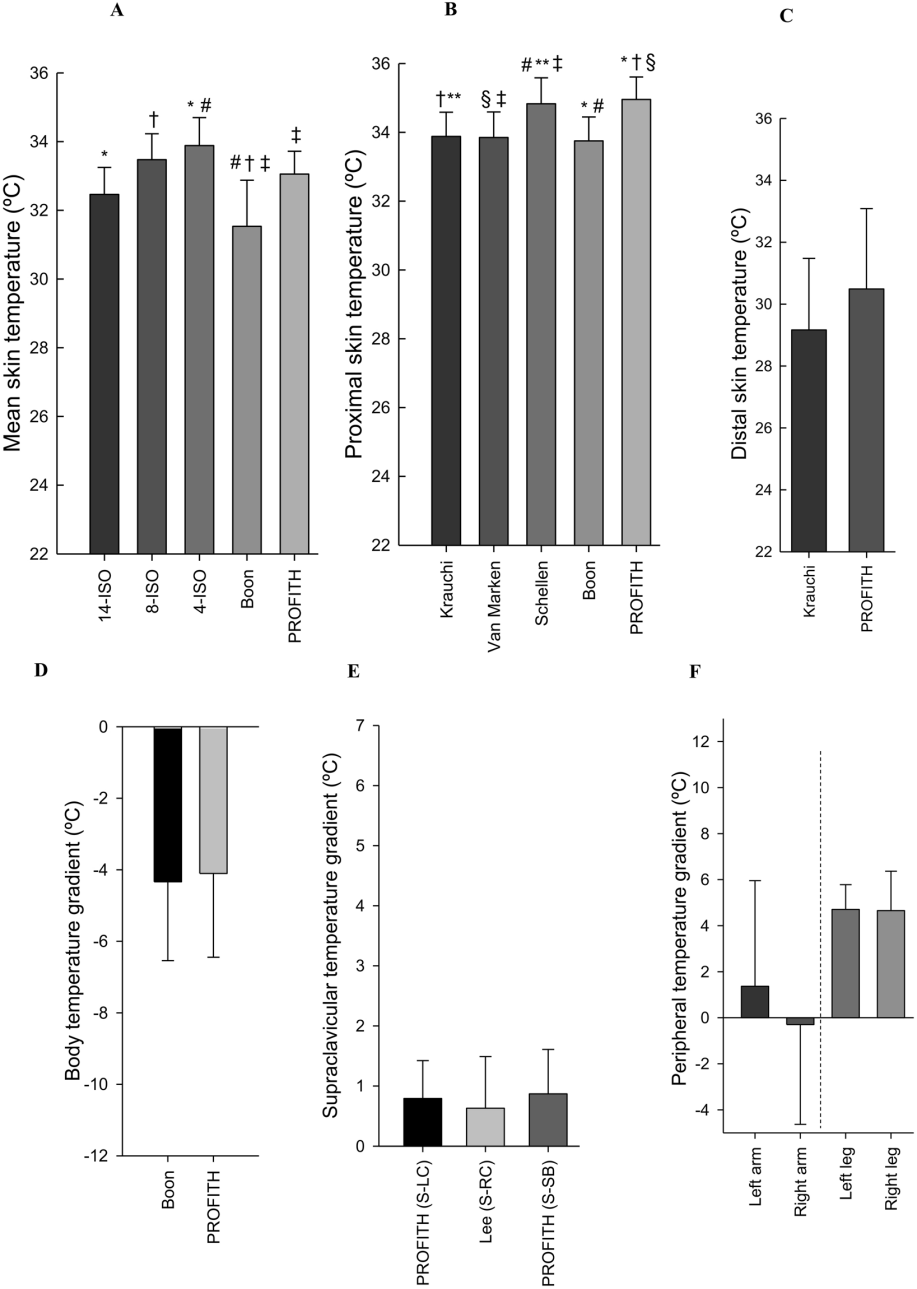
Measures of skin temperature at the last five minutes of the first warm period as estimated with the equations used in the respective references. (**A**) Mean skin temperature: *P = 0.022: 14-ISO vs. 4-ISO; ^†^P < 0.001: 8-ISO vs. Boon; ^#^P = 0.001: 4-ISO vs. Boon; ^‡^P = 0.021: Boon vs. PROFITH. (**B**) Proximal skin temperature: ^†^P = 0.015: Krauchi vs. PROFITH; **P = 0.048: Krauchi vs. Schellen; ^§^P = 0.011: Van Marken vs. PROFITH; ^‡^P = 0.036: Van Marken vs. Schellen; ^#^P = 0.014 Schellen vs. Boon; *P = 0.004: Boon vs. PROFITH. (**C**) Distal skin temperature: P = 0.222: Krauchi vs PROFITH. (**D**) Body temperature gradients: P = 0.931: Boon vs PROFITH. (**E**) Supraclavicular temperature gradients: (S: Supraclavicular; LC: Left Chest; RC: Right Chest; SB: Subclavicular); All P = 1.000. (**F**) Peripheral temperature gradient: All P = 1.000. Data are mean and standard error.

Figure 3 shows the mean skin temperature (A), proximal skin temperature (B), distal skin temperature (C), body temperature gradient (D), supraclavicular temperature gradient (E), and temperature gradients as a proxy for upper (left and right arm) and lower (left and right leg) peripheral vasoconstrictions (F) in the last five minutes of warm period I as estimated with the equations used in literature (Table 1). ANOVA showed differences across mean skin temperature equations (overall P < 0.001, Fig. 3A). The post-hoc analysis showed significant differences in mean skin temperature using the equation reported by 4-ISO^41^ compared with 14-ISO^41^ (mean difference 1.35 °C; 95% confidence interval: 0.12 °C–2.57 °C; P = 0.022) and between the equation reported by Boon *et al*.^15^ and 8-ISO^41^ (−1.83 °C; −3.02 °C–0.64 °C; P < 0.001), 4-ISO (−2.15 °C; −3.35 °C–0.96 °C; P = 0.001), and PROFITH (−1.40 °C; −2.67 °C–0.14 °C; P = 0.021).

Similarly, significant differences were observed across proximal skin temperature equations (overall P < 0.001, Fig. 3B). The post-hoc analysis revealed significant differences between the equation reported by Schellen *et al*.^32^ compared to Boon *et al*.^15^ (mean difference 1.03 °C; 95% confident interval: 0.13 °C-1.93 °C; P = 0.014), Kräuchi *et al*.^36^ (0.90 °C; 0.01 °C–1.81 °C; P = 0.048), and van Marken Lichtenbelt *et al*.^26^ (0.93 °C; −0.82 °C–1.84 °C; P = 0.036). We also observed significant differences between PROFITH and Boon *et al*.^15^ (1.16 °C; 0.26 °C–2.06 °C; P = 0.004), Kraüchi *et al*.^36^ (1.03 °C; 0.13 °C–1.93 °C; P = 0.015), and van Marken Lichtenbelt *et al*.^26^ (1.07 °C; 0.16 °C–1.96 °C; P = 0.011).

There were no significant differences between the equations used to estimate distal skin temperature (Fig. 3C), body (Fig. 3D), and supraclavicular (Fig. 3E) and peripheral (Fig. 3F) temperature gradients (all P ≥ 0.1).

### Cold period

**Figure 4:**
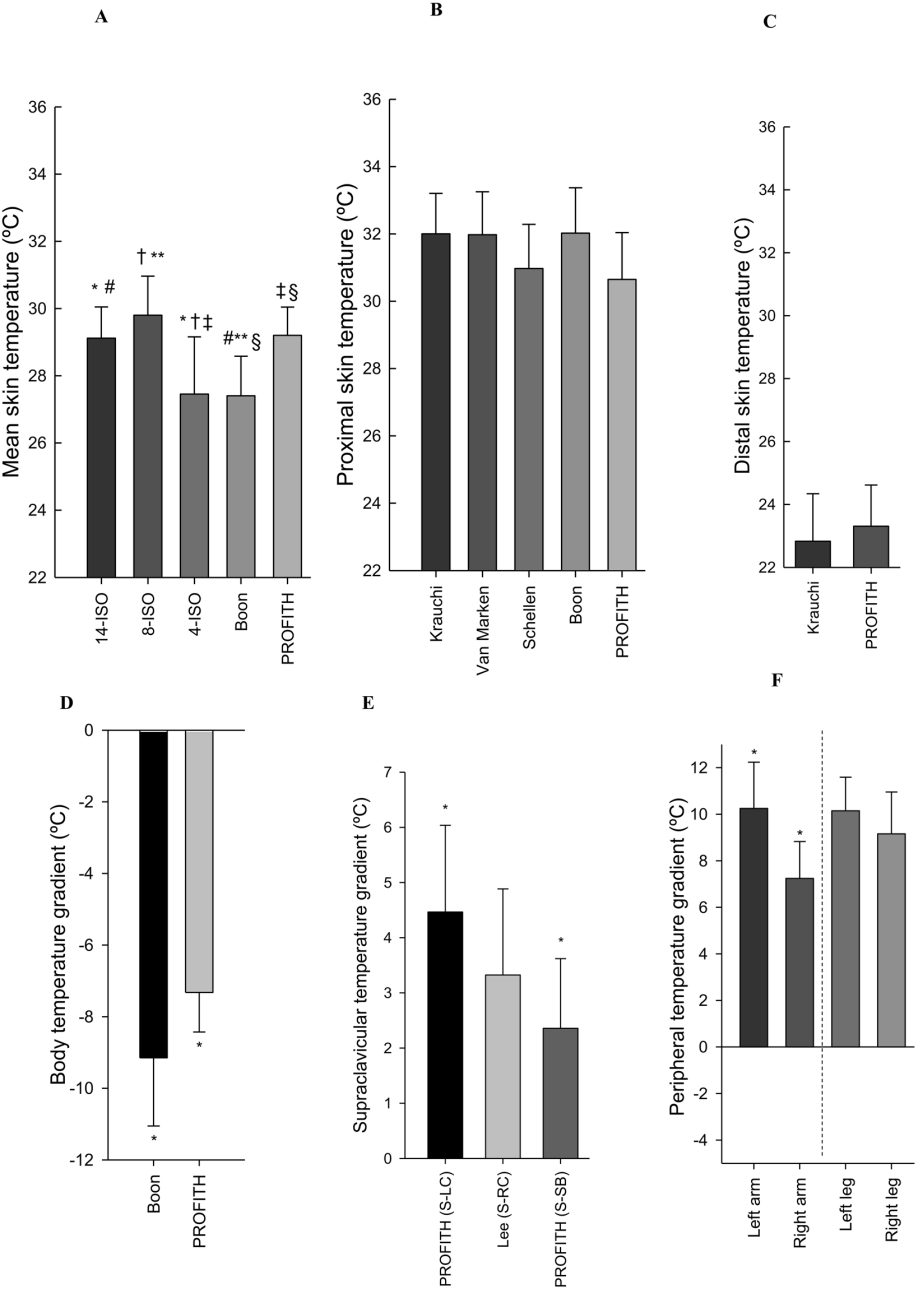
Measures of skin temperature at the last five minutes of the cold period as estimated with the equations used in the respective references. (**A**) Mean skin temperature: *P = 0.025: 14-ISO vs. 4-ISO; ^#^P = 0.027: 14-ISO vs. Boon; ^†^P ≤ 0.001: 8-ISO vs. 4-ISO; **P = 0.001: 8-ISO vs. Boon; ^‡^P = 0.022: 4-ISO vs. PROFITH; ^§^P = 0.024 Boon vs. PROFITH. (**B**) Proximal skin temperature: All P ≥ 0.123. (**C**) Distal skin temperature: P = 0.438. (**D**) Body temperature gradients: *P = 0.010 Boon vs. PROFITH. (**E**) Supraclavicular temperature gradients: (S: Supraclavicular; LC: Left Chest; RC: Right Chest; SB: Subclavicular) *P = 0.006; PROFITH (S-LC) vs. PROFITH (S-SB). (**F**) Peripheral temperature gradient: *P = 0.001; Left Arm vs. Right Arm. Data are mean and standard error.

Figure 4 shows the mean skin temperature (A), proximal skin temperature (B), distal skin temperature (C), body temperature gradient (D), supraclavicular temperature gradient (E), and temperature gradients as a proxy for upper (left and right arm) and lower (left and right leg) peripheral vasoconstrictions (F) in the last five minutes of the cold period as estimated with the various equations used in literature (Table 1). Differences across mean skin temperature equations were found in warm room conditions (overall P < 0.001, Figure 4A). The post-hoc analysis showed significant differences between the equation reported by Boon *et al*.^15^ and 14-ISO^41^ (mean difference −1.71 °C; 95% confident interval: −3.30 °C–0.12 °C; P = 0.027), 8-ISO (−2.36 °C; −3.90 °C–0.80 °C; P = 0.001), and PROFITH (−1.79 °C; −3.44 °C–0.15 °C; P = 0.024). Similarly, there were significant differences between 4-ISO^41^ and 14-ISO^41^ (−1.73 °C; −3.32 °C–0.14 °C; P = 0.025), 8-ISO^41^ (−2.37 °C; 0.83 °C–3.93 °C; P ≤ 0.001), and PROFITH (−1.81 °C; −3.45 °C–0.16 °C; P = 0.022).

There were no significant differences across equations used to estimate proximal (P = 0.123, Fig. 4B) and distal skin temperature (P = 0.438, Fig. 4C). There were, however, significant differences between the equations used to estimate the body temperature gradient (−1.82 °C; 95% confident interval: −0.82 °C− −2.83 °C; P = 0.01, Fig. 4D). Similarly, there were differences between the method to estimate supraclavicular temperature gradient zone when the skin temperature of the left chest zone was used instead of the right subclavicular zone (2.10 °C; 0.51 °C–3.70 °C; P = 0.006, Fig. 4E), as well as between the left and right gradient of the arms to estimate peripheral temperature gradients (3.01 °C; −0.97 °C −5.04 °C; P = 0.001, Figure 4 F).

### Warm period II

**Figure 5:**
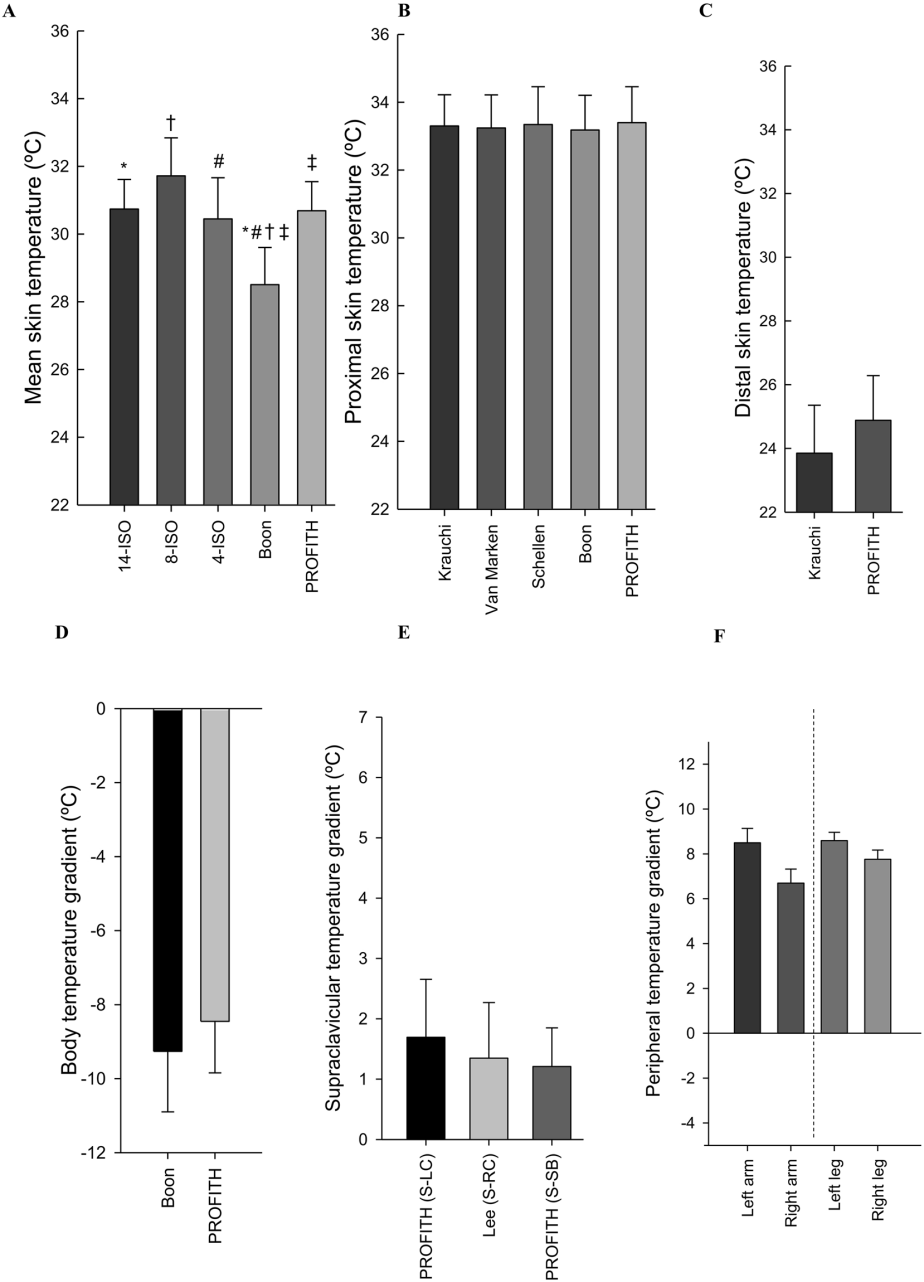
Measures of skin temperature at the last five minutes of the second warm period as estimated with the equations used in the respective references. (**A**) Mean skin temperature: *P ≤ 0.001: 14-ISO vs. Boon; ^†^P ≤ 0.001: 8-ISO vs. Boon; ^#^P = 0.002: 4-ISO vs. Boon; ^‡^P = 0.001: Boon vs. PROFITH. (**B**) Proximal skin temperature: All P = 1.000. (**C**) Distal skin temperature: P = 0.113. (**D**) Body temperature gradients: P = 0.252. (**E**) Supraclavicular temperature gradients: All P ≥ 0.575. (**F**) Peripheral temperature gradient: All P = 1.000. Data are mean and standard error.

Figure 5 shows the mean skin temperature (A), proximal skin temperature (B), distal skin temperature (C), body temperature gradient (D), supraclavicular temperature gradient (E), and temperature gradients as a proxy for upper (left and right arm) and lower (left and right leg) peripheral vasoconstriction (F) in the last five minutes of warm period II as estimated with the various equations used in literature (Table 1). Just as in warm period I, differences were found across mean skin temperature equations (overall P < 0.001, Figure 5A). The post-hoc analysis showed significant differences between the equation reported by Boon *et al*.^15^ and 14-ISO^41^ (mean difference −2.18 °C; 95% confident interval: −3.56 °C–0.79 °C; P ≤ 0.001), 8-ISO (−3.10 °C; −4.45 °C–1.76 °C; P ≤ 0.001), 4-ISO (−1.87 °C; −3.21 °C–0.52 °C; P = 0.002), and PROFITH (−2.12 °C; −3.55 °C–0.70 °C; P = 0.001). There were no significant differences (all P ≥ 0.1) between the equations used to estimate proximal (Fig. 5B) and distal skin temperature (Fig. 5C), as well as body (Fig. 5D), supraclavicular (Fig. 5E), and peripheral (Fig. 5F) temperature gradients.

## Discussion

Cold activates human BAT, which produces heat. Skin temperature is an indirect measure to monitor how the body reacts to cold. The present study analysed the impact of the most used equation in BAT-human studies to estimate parameters of skin temperature in warm and cold room conditions in young lean men. We observed differences across equations to measure the same parameters of skin temperature in warm and cold room conditions, which hamper comparisons across studies.

### Mean skin temperature

The equation reported by ISO STANDARD 9886:2004 using 14 ibuttons (14-ISO)^41^ is the most commonly used equation in BAT-related studies^7, 14, 18–23^, yet it has been used with substantial modifications^15, 24, 25^. Furthermore, studies report values of mean skin temperature without providing information on how the calculations were made^12, 17, 29^, which hampers between-study comparisons. Mean skin temperature estimated with 14-ISO was similar to that estimated using 26 ibuttons (PROFITH equation, see Table 1), which suggests that the 14-ISO equation covers the most important body sites. The ISO STANDARD 9886:2004 suggested another set of 8 ibuttons (8-ISO)^41^ to measure mean skin temperature. However, the 8-ISO slightly overestimates temperature in both warm and cold conditions, at least when compared with the other equations used in this study.

Similarly, the equation based on 4 ibuttons (4-ISO)^41^ overestimates mean temperature in warm room conditions and underestimates mean temperature in cold conditions compared to 14-ISO. The anatomical sites used in 4-ISO may partially explain the observed differences. For instance, the temperatures of the shin bone and the hand decreased after cold exposure^14^ probably due to peripheral vasoconstriction, while these two anatomical zones contribute 50% of the estimated mean temperature in the equation 4-ISO. Based on these findings, we suggest using the 14-ISO equation to measure mean skin temperature because (i) it is the most used equation in BAT-human studies, (ii) it is supported by the International Standard Organization, and (iii) the outcome of temperature is practically the same when it is compared with an equation with a higher number of ibuttons.

### Proximal skin temperature

We did not observe differences between the equations used to estimate proximal skin temperature during cold exposure or during the warm period II. On the other hand, there were differences between the study equations during the warm period I. The equations reported by Kräuchi *et al*.^36^ and Schellen *et al*.^30^ used four ibuttons while the equations reported by van Marken Lichtenbelt *et al*.^26^ and Boon *et al*.^15^ used three ibuttons. The equations reported by both van Marken Lichtenbelt *et al*.^26^ and Boon *et al*.^15^ are based on the same anatomical points. However, Boon *et al*.^15^ used the clavicular zone whereas van Marken Lichtenbelt *et al*.^26^ used the subclavicular zone (Table 1). These equations showed a similar decrease of the mean temperature after cold exposure: −1.72 ± 0.85 °C^15^ and −1.87 ± 0.76 °C^26^, respectively. To assess proximal skin temperature, we suggest the equation reported by Boon *et al*.^15^ as it includes a button at the clavicular site which is close to BAT deposits. In addition, the outcome of proximal temperature could be more representative from a body reaction to cold than other equations.

### Distal skin temperature

To measure distal skin temperature, the majority of BAT-related studies^7, 14, 18–23^ used the equation reported by Kraüchi *et al*.^36^ (2 ibuttons placed on the left hand and right instep). Indeed, this equation is one of the easiest to estimate distal skin temperature. We did not observe differences between distal skin temperature measured by this and other equations with a higher number of distal anatomical points (6 ibuttons, see PROFITH, Table 1) in warm or cold conditions (all P ≥ 0.113). In addition, the 14 anatomical points recommended by ISO STANDARD 9886:2004^41^ include the anatomical positions used by the equation reported by Kraüchi *et al*.^36^. Thus, under these study conditions, the equation reported by Kraüchi *et al*.^36^ is a valid choice to estimate distal skin temperature. Moreover, two distal ibuttons (hand and feet) can measure the same as other equations with a higher number of ibuttons (i.e. 6 devices in PROFITH equation, see Table 1).

### Whole body temperature gradients

The heat loss capacity of the body can be estimated as the gradient between distal and proximal skin temperature. Such gradients have been used in various fields including circadian rhythm^34, 36, 42^, anaesthesia^24^, exercise^27, 43, 44^, and following BAT activation^7, 14, 18–23^. We observed no differences between the gradients calculated by the equation reported by Boon *et al*.^15^ (5 ibuttons) and other equations with a higher number of anatomical points (11 ibuttons, see PROFITH, Table 1). Therefore, we suggest to use the equation reported by Boon *et al*.^15^ as it obtains the same outcome of temperature with a lower number of ibuttons than other equations.

Another interesting gradient that has recently been used in the field of BAT research is the supraclavicular temperature gradient proposed by Lee *et al*.^16^. This gradient is based on the difference between the temperature of the right supraclavicular fossa and right upper chest and aims to estimate BAT heat loss capacity during or after a cold exposure. Yoneshiro *et al*.^12^ and Chondronikola *et al*.^17^ used the same gradient albeit at the left side of the body. The importance of the side of the body is unknown and further studies are warranted. Nevertheless, we observed differences when the supraclavicular temperature gradient is calculated on the right or the left side, as well as on the chest or the subclavicular zone. We propose to use the supraclavicular temperature gradient reported by Lee *et al*.^16, 29^. This gradient was validated against ^18^F-FDG-PET/CT and infrared thermography in 87 lean individuals^29^ while the validity of other gradients has not yet been proved^12, 17^.

**Figure 6:**
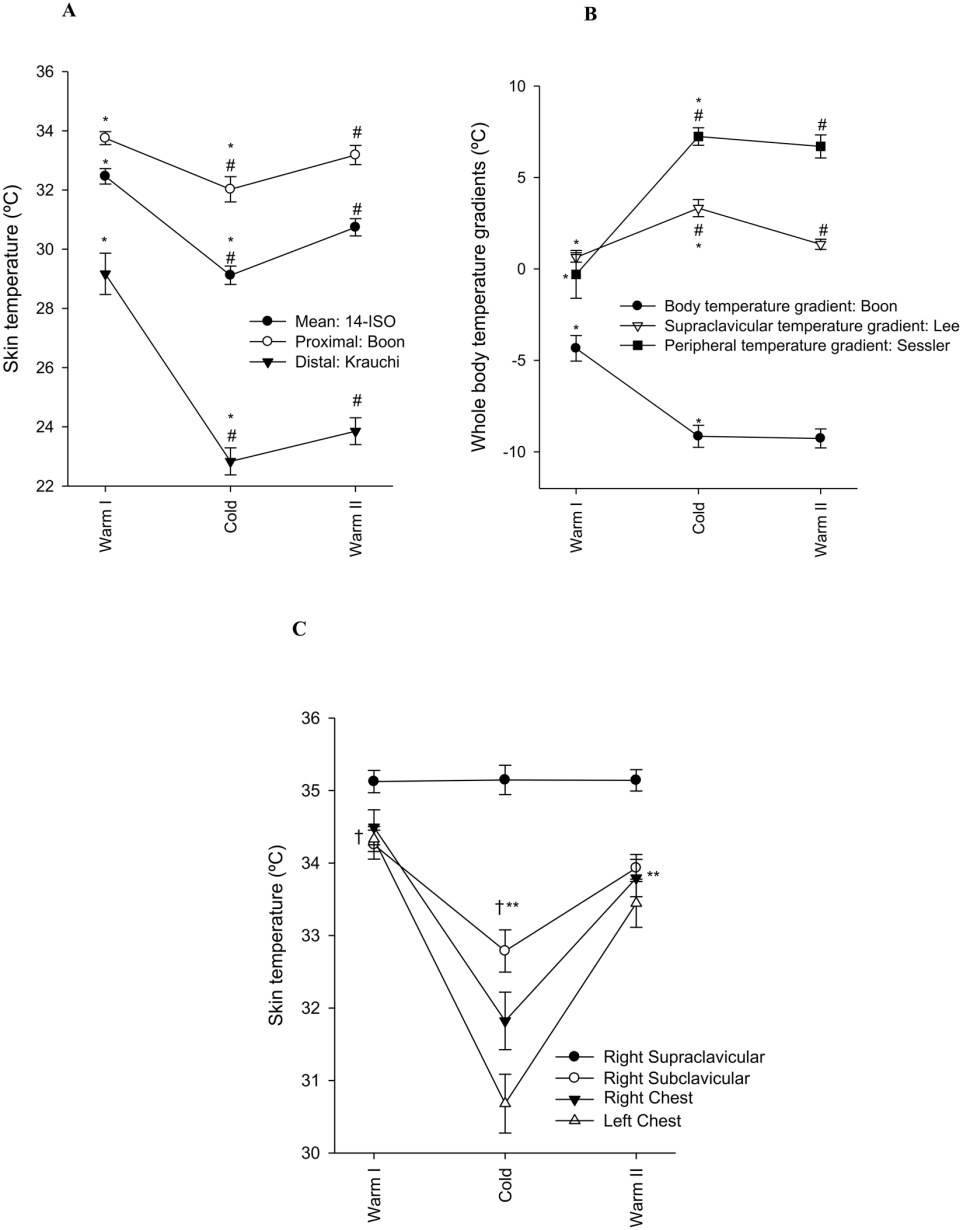
Suggested equations to measure different parameters of skin temperature during warm and cold exposures. (**A**) Mean skin temperature (14-ISO): *P ≤ 0.001: Warm I vs. Cold; ^#^P ≤ 0.001: Cold vs. Warm II; ^#^P = 0.002. Proximal skin temperature (Boon): *P ≤ 0.001: Warm I vs. Cold; ^#^P ≤ 0.001: Cold vs. Warm II. Distal skin temperature (Krauchi): *P ≤ 0.001: Warm I vs. Cold; ^#^P ≤ 0.001: Cold vs. Warm II. (**B**) Body temperature gradient (Boon): *P ≤ 0.001: Warm I vs. Cold. Supraclavicular temperature gradient (Lee): *P ≤ 0.001: Warm I vs. Cold; ^#^P ≤ 0.001: Cold vs. Warm II. Peripheral temperature gradient (Sessler): *P ≤ 0.001: Warm I vs. Cold. (**C**) Raw data of all anatomic points integrated in the equations to estimate supraclavicular temperature gradients: all ^†^P≤0.001: Warm I vs. Cold; all **P ≤ 0.001: Cold vs. Warm II. No significant differences were found in the supraclavicular skin temperature across the exposures. Data are mean and standard error.

Interestingly, we identified the right supraclavicular skin temperature as the only marker that did not decrease during cold exposure (Fig. 6), which is in line with the hypothesis that cold exposure activates BAT, and that BAT generates heat. Besides, this region was properly covered by the cooling vest. Although we have no data on BAT activity and volume of the participants, this finding concurs with other studies that showed that supraclavicular skin temperature was positively associated with BAT activity and volume in the supraclavicular zone in healthy young men^12, 14, 15, 17, 45^. However, we cannot ignore that the absence of supraclavicular skin temperature decrease upon cold exposure is due to the presence of large blood vessels (i.e. aorta) close to the skin in this area. More studies are required to confirm this finding, as well as to elucidate the role of body mass index and subcutaneous adipose tissue in the measurement of all parameters of skin temperature^45–48^.

Peripheral temperature gradient is a proxy of peripheral vasoconstriction. This gradient has been used as a marker for changes in the blood flow in peripheral zones. The peripheral vasoconstriction is a strategy of the body to keep the organs warm during cold exposure^5^ carrying some of the peripheral blood to the central part of the body. House & Tipton^37^ proposed a gradient between the temperature of the right top of the forefinger and the temperature of the middle part of the right forearm. They validated this gradient against laser Doppler flowmetry and reported that a difference of 2 °C may indicate peripheral vasoconstriction. In contrast, a gradient lower than 0 °C suggests peripheral vasodilatation. Sessler *et al*.^24, 49^ reported vasoconstriction by a difference of ≥4 °C, while a difference lower than 4 °C points to peripheral vasodilatation. Of note is that both thresholds are reached in our experimental conditions. However, other studies used the same peripheral temperature gradient as a proxy of peripheral vasoconstriction but in different anatomical positions^14^ or calculated the gradient in a different way^25^. The best way to estimate peripheral vasoconstriction is currently not known and further studies are warranted. We calculated the same gradient but in the lower part of the body (instep-gastrocnemius) and observed an increase of this gradient after cold exposure. Therefore, a peripheral vasoconstriction occurs in the lower part of the body, as it happens in the upper part of the body, as previously reported^14, 50^.

### Limitations

Results of this study should be considered with caution. Data are based on a single cooling protocol, using a cooling vest (that covers only chest and back zones), and we do not know whether the results apply to different cooling protocols or instruments (e.g. cooling blankets or ice blocks) or to longer cold exposure after shivering occurs. Additionally, the study was conducted on young lean men and we do not know whether these results apply to older people, to women, or to persons with higher (or lower) levels of body fat or in narrower ranges of hours. This study was performed in the south of Spain, and the equations were used under a personalised cooling protocol. Therefore, we are unaware if the results match those of other countries or environments and whether they can differ under warming or exercise protocols. We cannot ignore that the time of the day when our study was conducted may have influenced the results. Due to the methodological nature of this study, our findings are not comparable with other studies because they have used other cooling or warming protocols^7, 14, 18–23^.

## Conclusion and Recommendations

We detected differences in skin temperature across the studied equations in both warm and cold room conditions. Based on these findings, we suggest a set of 19 ibuttons to estimate mean, proximal, and distal skin temperatures as well as body temperature gradients. We recommend to measure mean skin temperature with the 14-ISO equation^41^; proximal and body gradient of skin temperature with the Boon *et al*. equation^15^; distal skin temperature with the Krauchi *et al*. equation^36^; supraclavicular temperature gradient with Lee *et al*. equation^16, 29^ and peripheral temperature with Sessler *et al*. equation^3^ (Table 3). These equations are based on the 14 anatomical positions reported by ISO STANDARD 9886:2004^41^ plus five more ibuttons placed on the right supraclavicular fossa, right middle clavicular bone, right middle upper forearm, right top of forefinger, and right upper chest (Fig. 1: ibuttons 1–16, 20, 21, and 25, respectively). Moreover, we have seen that all selected equations are sensitive to the cooling protocol study (see Fig. 6), except the supraclavicular skin temperature which was similar across temperature conditions.

**Table 3:** Recommended equations to measure skin temperature.

Outcome	Reference	ibuttons (n)	Anatomical positions (Fig. 1)	Equation	Rationale to select the equation
Mean skin temperature	14 ISO 9886–2004^41^ *(14-ISO)*	14	From 1 to 14	(Forehead*0.07) + (Neck*0.07) + (Right Scapula*0.07) + (Left Chest*0.07) + (Right Deltoid*0.07) + (Left Elbow*0.07) + (Right Abdomen*0.07) + (Left Hand*0.07) + (Left Lumbar *0.07) + (Right Thigh*0.07) + (Left Hamstring*0.07) + Right Shinbone*0.07) + (Left Gastrocnemius*0.07) + (Right Instep*0.07)	Mean skin temperature estimated with 14-ISO was similar to that estimated using 26 ibuttons (PROFITH equation), which suggests that the 14-ISO equation covers the most important body sites with less ibuttons.
Proximal skin temperature	Boon *et al*.^15^ *(Boon)*	3	10, 16, 8	(Right Thigh*0.383) + (Right Clavicular*0.293) + (Right Abdomen*0.324)	This equation includes less ibuttons than other equations, and it also includes an ibutton at the clavicular site which is close to BAT deposits.
Distal skin temperature	Kräuchi *et al*.^36^ *(Krauchi)*	2	9, 14	(Left Hand + Right Instep)/2	Distal skin temperature estimated with this equation was similar to that estimated using 6 ibuttons (PROFITH equation), which suggests that this equation covers the most important body sites with less ibuttons.
Body temperature gradient	Boon *et al*.^15^ *(Boon)*	5	9, 14, 10, 16, 8	[(Left Hand + Right Instep)/2] − [(Right Thigh*0.383) + (Right Clavicular*0.293) + (Right Abdomen*0.324)]	Body temperature gradient estimated with this equation was similar to that estimated using 11 ibuttons (PROFITH equation), which suggests that this equation covers the most important body sites with less ibuttons.
Supraclavicular temperature gradient	Lee *et al*.^29^ *(Lee S-RC)*	2	15, 25	(Right Supraclavicular(S) - Right Chest (RC))	This equation has been validated against ^18^F-FDG-PET/CT and infrared thermography.
Peripheral temperature Gradient	Sessler *et al*.^3^ Right arm	2	20, 21	(Right Forearm-Right Top of forefinger)	This equation has been validated against laser Doppler flowmetry.
